# Surgery

**Published:** 1904-06

**Authors:** C. A. Morton


### SURGERY.

Mr. P. J. Freyer has now published a large number of very successful cases of **Prostatectomy**, but a series of papers recently published[1] by other surgeons with reference to Freyer's operation are also of interest. Mr. Barling, of Birmingham, records ten cases, with three deaths, but only one death could really have been caused by the operation, and that was from uræmia in the third week after operation. Mr. Moynihan publishes twelve cases with one death. The immediate result of the operation in Mr. Barling's cases was very good, and in some cases examined many months after the operation great improvement was maintained. In one case slight incontinence at night occurred, but this was quite an exception. Mr. Moynihan gives so little account of the condition of the patients after operation that the value of his paper is greatly diminished. All he states is when the wounds healed. Mr. Barling found that if the enlargement was considerable and the gland elastic, enucleation might be easily accomplished, but that in the firmer variety of prostatic enlargement it may require all the force of which the fingers are capable. Mr. Moynihan writes of the operation that the enucleation is easily performed, and that the larger the prostate the easier the stripping. In one case in which I enucleated a large prostate weighing four ounces, it shelled out easily in some parts, but in others required considerable force to dislodge it; in another case in which it was much smaller, after using considerable force I had not succeeded in entirely separating it, and as I did not care to use more force, I contented myself with cutting off a projecting collar of intravesical growth. The patient had been unable to pass any urine for some time before operation, and catheterisation was often difficult. His condition has been greatly improved by the operation, and he now passes urine but with a feeble stream. No doubt complete prostatectomy would have given a better result. I had been so impressed in this case by the fact that great force may be required to shell out a prostate only moderately enlarged, but which is yet causing retention, that in another case on which I operated soon after I enucleated simply a projecting so-called "middle lobe" which was acting as a ball valve obstruction, and thus enabled the patient to pass urine, but not to empty the bladder. In two of the cases stones were detected in the bladder before operation, so that the operation was a lithotomy as well as a prostatectomy. In the case in which I removed the very large prostate, some incontinence followed, but his condition was very greatly improved. It is interesting to note that one of Mr. Barling's patients, from whom he removed an enlarged prostate which was composed almost entirely of overgrowth

[1] Barling, *Brit. M. J.*, 1904, i. 232; Wallace, *Ibid.*, 239; Moynihan, *Ann. Surg.*, 1904, xxxix. 1; Walker, *Lancet*, 1904, i. 869.

of connective tissue, and which had made the patient entirely dependent on the use of the catheter, was only 46 years of age, and Mr. Barling states that he has had other cases of prostatic enlargement in men just over 40. Another of Mr. Barling's cases is also remarkable, as although the patient (a man aged 60) had been dependent on the catheter for many weeks, yet the prostate after removal weighed very little more than normal, though it had felt moderately enlarged and very hard per rectum. The enucleation, Mr. Barling tells us, was very difficult owing to its firm fixation to adjacent part. Bleeding does not seem to have been serious in Mr. Barling's cases. He thinks that from four to six ounces of blood is usually lost during the enucleation, which must proceed in spite of the oozing, but that when the prostate has been freed this lessens, and what remains can be readily controlled by sponge pressure or hot douching. Mr. Moynihan says that in his cases as a rule there was very little bleeding, but that in two there was free hemorrhage for twenty to thirty minutes. In a case of my own, to which I have already referred (removal of large prostate), the hemorrhage was very free indeed, and required prolonged sponge pressure and hot douching to check it.

Mr. S. Shattock has shown from an examination of some of the specimens of enlarged prostate removed by Mr. Freyer that though it was thought that the prostatic urethra had not been removed it was there in his specimens, but so thin and spread out over the adenomatous masses as only to be recognised by microscopic section; and it became evident that what had been supposed to be the prostatic urethra torn away with some of the prostates was really a part of the membranous urethra, and both Mr. Barling and Mr. Moynihan regard removal of the prostatic urethra as an almost necessary part of the operation. Mr. Barling, however, maintains that no stricture follows this tearing out of the prostatic uretha, and occasionally part of the membranous urethra, and the later records of cases published by Freyer and others seem to justify the belief.

Whether the whole prostate is removed in this operation, as Mr. Walker has lately maintained [1] in a paper read at the Royal Medico-Chirurgical Society, based on an examination of seventy of Freyer's specimens, or that a capsule of prostatic tissue is always left behind, as Wallace endeavoured to prove in a paper read at a meeting of the Pathological Society last winter,[2] it seems difficult to say. Possibly, as suggested by Reginald Harrison[3] in some cases in which the enucleation is easy, adenomatous masses (generally including the prostatic urethra) are shelled out of a capsule of flattened-out prostatic tissue, whereas in the more difficult cases the whole gland is shelled out from the sheath of recto-vesical fascia. Not only have Mr. Walker and Mr. Wallace examined many specimens of prostates removed by Freyer's method, but they have also

[1] *Loc. cit.* [2] *Loc. cit.* [3] *Lancet*, 1904, i. 869.

examined what is left behind after removal of the prostate, and this one would think ought to settle the vexed question. Mr. Walker has done so in four cases, and he reports [1] that "the prostatic urethra was left behind entirely in one, partly in one, and completely removed in two. The posterior wall of the cavity (from which the prostate had been removed) was formed at the upper part by the seminal vesicles enclosed in the recto-vesical fascia. This fascia was continued downwards to form the lower part of this wall. A layer of unstriped muscle was continued downwards from the bladder wall at the upper part of the circumference of the cavity. The lateral walls were formed by the pelvic fascia, outside which were the levator ani muscles. The anterior wall consisted of layers of fascia, which enclosed between them the vertical part of the prostatic plexus." In one case a small adenomatous nodule was found left behind after the enucleation. Mr. Wallace enucleated an enlarged prostate in a dead body exactly as the operation is performed in the living, and found that a shell of expanded prostatic tissue was left behind.

There is one very remarkable specimen figured in Wallace's paper of a prostate removed from a man of 65, for "dribbling retention." It is certainly not an enlarged prostate. That the operation in this case could have consisted in the shelling out an adenomatous mass or masses from a capsule of prostatic tissue seems obviously impossible; the specimen suggests that a prostate of normal size (5 drachms in weight) was enucleated.

In the lectures on the **Surgery of the Pancreas**, recently delivered at the College of Surgeons by Mr. Mayo Robson, are some statements of much practical importance. Mr. Robson tells us [2] that by a chemical examination of the urine it is possible to diagnose the presence of disease of the pancreas, and even to distinguish between malignant disease and chronic inflammation of the gland. We are told that by this reaction in the urine acute pancreatitis, which has in the past been so often mistaken for intestinal obstruction, may be differentiated, and several cases are recorded to illustrate the value of the test in distinguishing between malignant disease of the head of the pancreas causing obstructive jaundice by pressure on the common duct, and induration of the head from chronic inflammation acting in the same way, or from gallstones in the common duct. Two cases are recorded in which a diagnosis of malignant disease was corrected by the application of the test, and operation which was thus seen to be indicated was successfully performed.

The test has been worked out by Dr. Cammidge, and is fully described by him in his lecture delivered before the Royal College of Surgeons on the chemistry of the urine in disease of the pancreas.[3] The test depends on the presence of minute

[1] *Loc. cit.* [2] *Lancet*, 1904, i. 780. [3] *Lancet*, 1904, i., 782.

quantities of glycerine in the urine in pancreatic disease, and by the action of chemical reagents crystals are obtained of diagnostic significance. We must refer readers to the lecture for the minute details of the process. That certain substances should be present in the urine if the functions of the pancreas are imperfectly performed, or if any of its secretion should escape and act on the tissues is not more than we might expect, but that by a chemical test of the urine we should be able to distinguish between the diseases which impair the function or cause such escape of secretion—between acute and chronic pancreatitis and between the latter and malignant disease—seems almost incredible, especially when we find that it is the time which the crystals take to dissolve in dilute sulphuric acid which enables such differentiation to be made. Yet Mr. Mayo Robson seems to have derived very great assistance in diagnosis from the work of Dr. Cammidge. We should, however, be very reluctant to trust to the diagnosis of those who had not done considerable work in the application of this test.[1]

Very little has as yet been written about pancreatic calculi, and therefore the information given by Mr. Robson on this subject is of sufficient importance to demand a brief reference. As is well known, such calculi may give rise to symptoms very like those of biliary colic, but the pain tends to radiate to the angle of the left scapula, and they are generally associated with other diseases of the gland—chronic inflammation, cysts, or abscess—and are usually multiple. An important fact pointed out by Mr. Robson is that, unlike gallstones, they may be detected in a skiagram, as they contain lime salts. They may pass into the diverticulum of Vater and cause obstructive jaundice, and become covered with cholesterine and stained with bile, so that they may resemble a gallstone. After a thorough search through English and foreign literature, Mr. Robson could only find five cases of removal of these pancreatic calculi. If the stone lies in the main pancreatic duct just at its junction with the common duct it may be reached by first opening the duodenum, and then the opening of the common duct enlarged and the stone extracted from the duct of Wirsung. Or a stone in the pancreas may be removed through either the gastro-hepatic or the great omentum, by direct incision into the pancreas, followed by careful suture of the duct and divided pancreatic tissue. Sometimes it is suggested the stone may be

[1] Since the above was written Dr. C. Ham and Dr. J. Burton Cleland have written from the Pathological Institute of the London Hospital, and Dr. Grenner from his Pathological Laboratory, Leeds Infirmary, stating that they have found the crystals described by Dr. Cammidge present in almost any urine (*Lancet*, 1904, ii. 1378). Dr. Cammidge maintains that the crystals they have produced are not the same as those he has obtained in pancreatic disease, and that their method is in fault. It is as I stated above quite clear that if his test is reliable it can only be carried out by one as familiar with it and its possible sources of error as Dr. Cammidge.

better reached by dividing the reflection of peritoneum over the second part of the duodenum, and thus gaining access to the head of the pancreas either on the inside of the second portion of the duodenum or from behind it.

There is another fact of such importance in Mr. Robson's lectures that we must also refer to it. From his own experience and that of Murphy of Chicago it seems quite certain that no good is to be gained by draining the gall-bladder in jaundice due to pressure of the cancerous head of the pancreas on the common duct, for the mortality of the proceeding is very high, and if recovery from the operation occurs life is not prolonged to any great extent.[1] At the same time it must be pointed out that chronic inflammatory induration of the head of the pancreas is now well known to cause symptoms resembling those of malignant disease of the head of the pancreas, and that in cases of doubt exploration is indicated, for recovery has often followed drainage of the gall-bladder in the former condition. But Mr. Robson now considers that exploration is hardly required, as the chemical examination of the urine ought to enable us to decide between these two conditions.

C. A. MORTON.

